# Supplementary material for: Robust edge photocurrent response on layered type II Weyl semimetal WTe2
Source: Nat Commun. 2019 Dec 16;10:5736. doi: 10.1038/s41467-019-13713-1 (PMC6915719; doi:10.1038/s41467-019-13713-1)
Supplement: Supplementary file 1 — Supplementary Information [file 41467_2019_13713_MOESM1_ESM.pdf]

Robust Edge Photocurrent Response on layered type II Weyl Semimetal WTe_2

Wang et al.

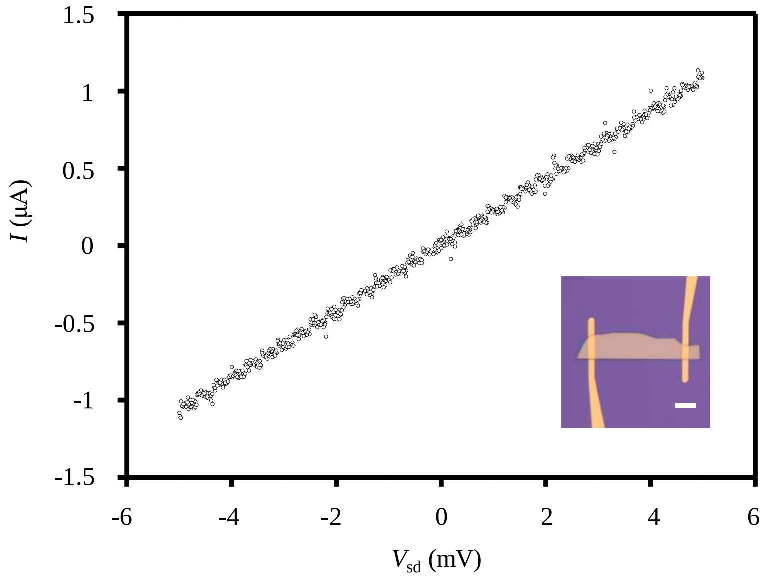

Supplementary Figure 1. *I-V* Characteristic of the WTe_2 device used in Figure 2a. The bias voltage was varied from -5 mV to 5 mV with a step size of 50 μV, and the drain current was converted to voltage with a low noise current amplifier. The *I-V* characteristic shows clear linear dependence of the drain current on the bias voltage, which indicates good Ohmic contacts between the WTe_2 flake and the electrodes. The two-terminal resistance of this device is 4.2 KΩ. The inset is the optical microscope image of the device. The scale bar is 8 μm.

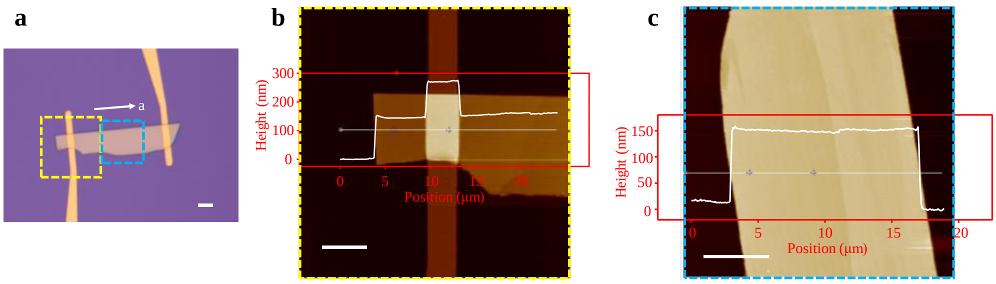

Supplementary Figure 2. AFM images of the WTe_2 device used in Figure 2a. (a) Optical microscope image of the device. (b), (c) Atomic force microscope (AFM) images of areas marked by the yellow (b) and blue (c, with 90-degree rotation) square boxes in panel a, with a scanning area of 30 μm * 30 μm and 20 μm * 20 μm respectively. The red boxes show sectional profiles of the white lines marked in the AFM images. Several-nm height steps were observed on the scanning areas of the

WTe_2 flake as identified by the AFM images in Supplementary Figure 2b, c. The thicknesses of the WTe_2 devices were measured with AFM (Bruker multimode8) under tapping mode. The thickness of WTe_2 flake is about 150 nm, with 100-nm thick metal contacts on it. The scale bars are all 5 μm .

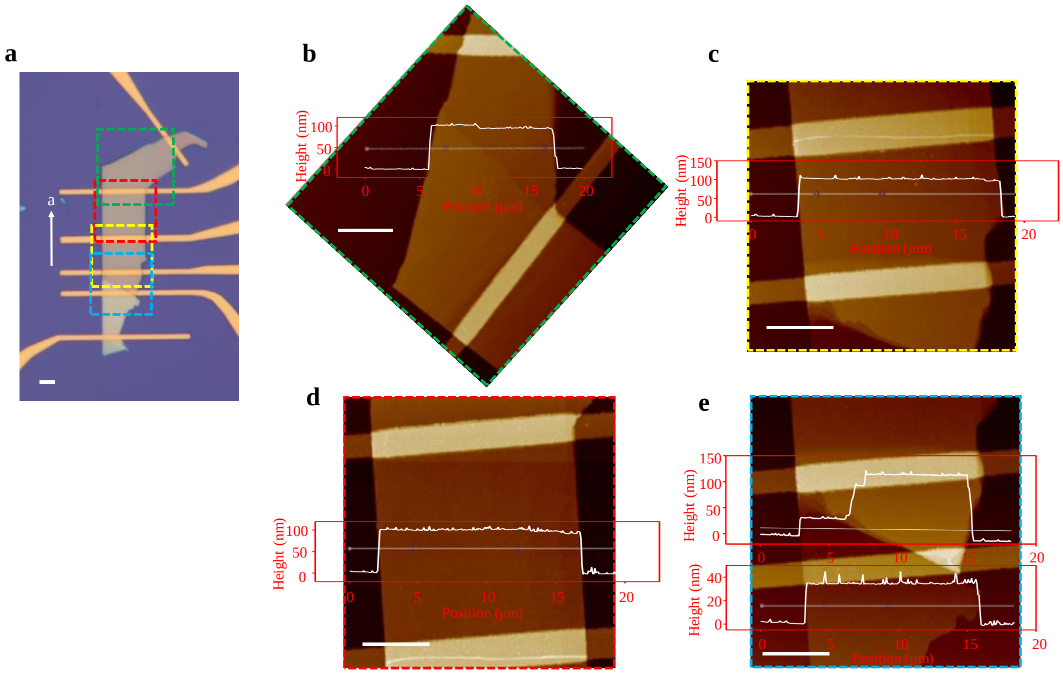

Supplementary Figure 3. AFM images of the WTe_2 device used in Figure 2e. (a) Optical microscope image of the device. (b)-(e) AFM images of the areas marked by green (b with 45-degree rotation), yellow(c), red(d) and blue(e) square boxes in Figure a, with a scanning area of 25 μm * 25 μm in Supplementary Figure 3b, and 20 μm * 20 μm in Supplementary Figure 3c-e. The red boxes show sectional profiles of the white lines marked in the AFM images. The insets in Supplementary Figure 3b-d are the sectional profiles of the white lines in the images. The scale bars are all 5 μm.

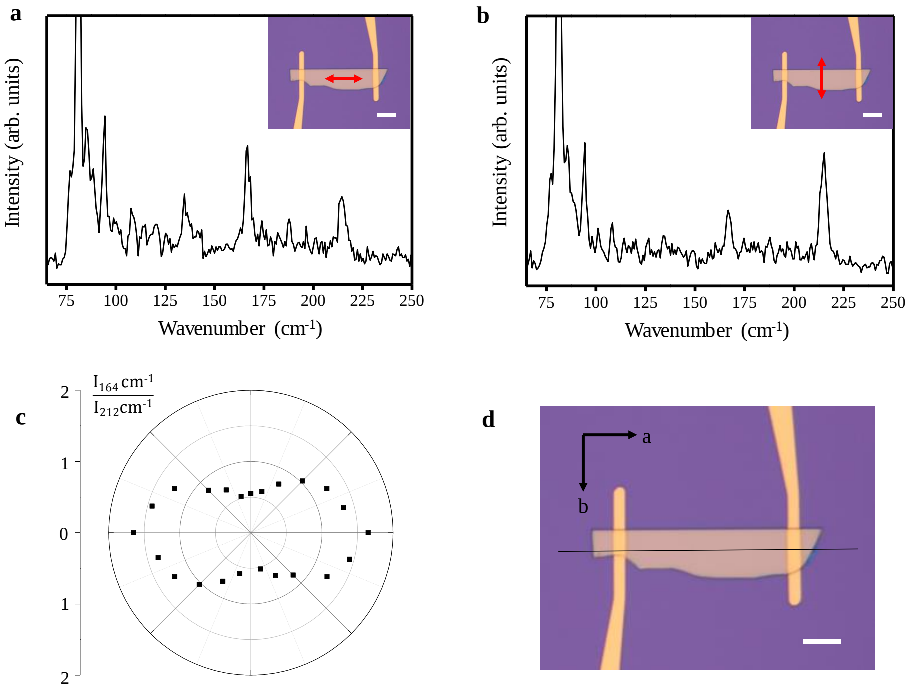

Supplementary Figure 4. Polarization resolved Raman spectroscopy of WTe_2. (a) Raman spectroscopy with the excitation polarization parallel to the long edge of the WTe_2 flake used in Figure 2a. (b) Raman spectroscopy with the excitation polarization perpendicular to the long edge. (c) The intensity ratio of Raman peaks 164 cm^{-1} and 212 cm^{-1} at different excitation polarization. The ratio reaches maximum when the laser polarization is along the direction of long edge. (d) Optical microscope image of the device. The scale bars are all 8 μm.

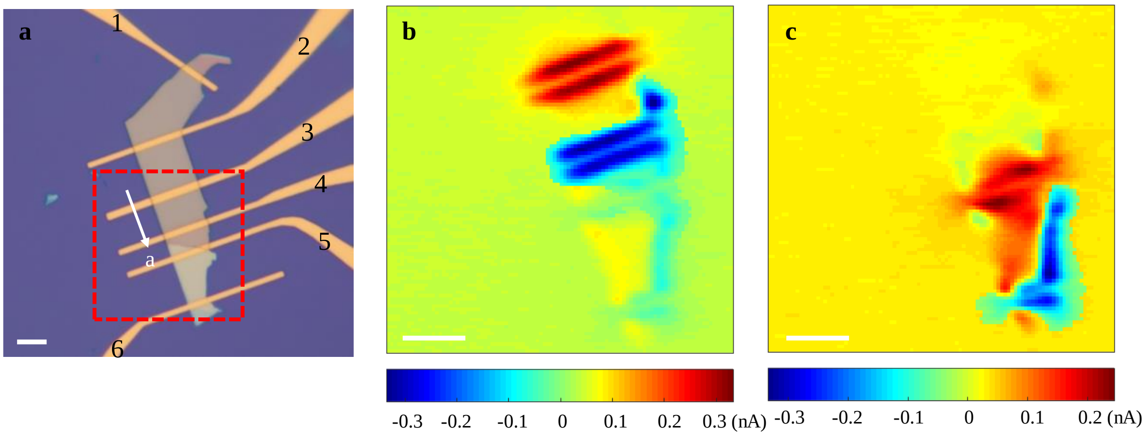

Supplementary Figure 5. Scanning photocurrent microscopy results. (a) Optical microscope image of the WTe_2 device used in Figure 2e. (b), (c) Scanning

photocurrent microscopy (SPCM) results of the rectangular area marked by the red box in Figure a. The measurements were performed using contacts 3 and 4 (b), and contacts 5 and 6 (c), respectively. All measurements were performed with 180-μW 1.96 eV pulse excitation. The scale bars are all 8 μm.

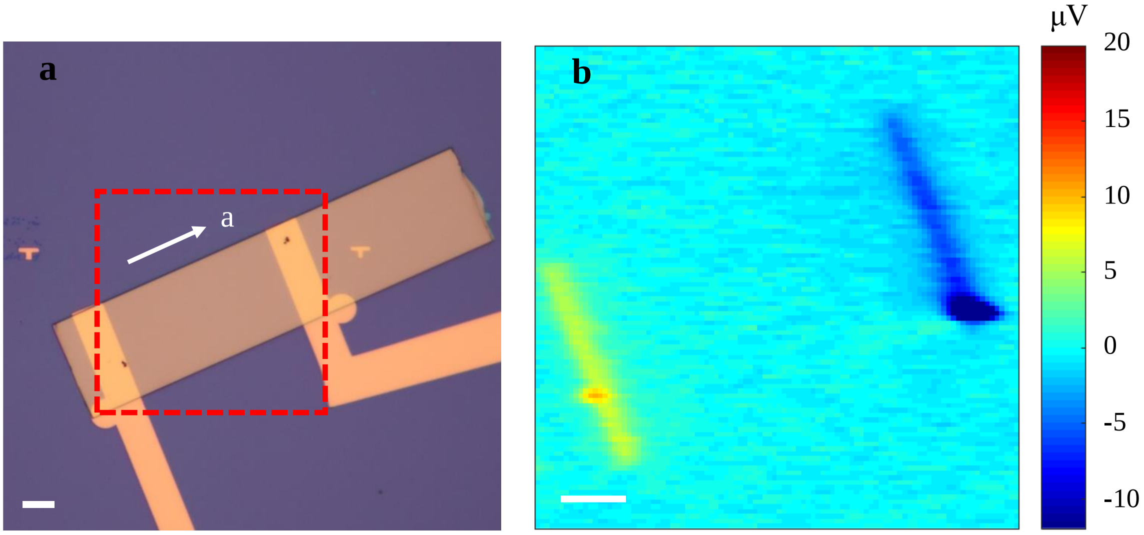

Supplementary Figure 6. Scanning photovoltage microscopy result. (a) Optical microscope image. (b) Scanning photovoltage microscopy result of the rectangular area marked by the red box in Figure a. The measurement was performed with 1.38-mW 1.55-eV pulse excitation. The scale bars are all 10 μm.

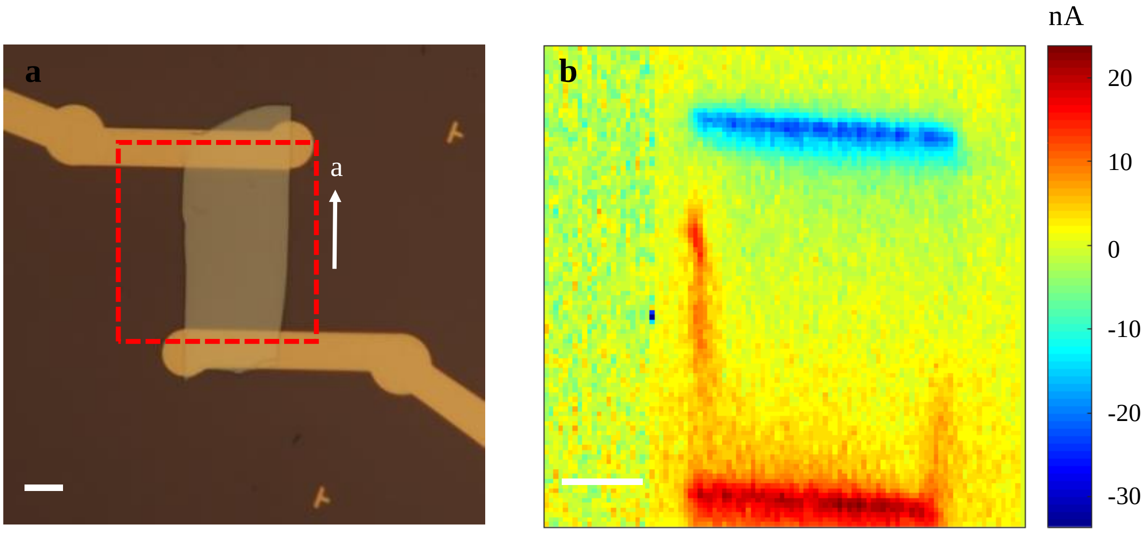

Supplementary Figure 7. Scanning photocurrent microscopy result. (a) Optical microscope image. (b) Scanning photocurrent microscopy result of the rectangular area marked by the red box in Figure a. The measurement was performed with

300-μW 1.55-eV continuum wave excitation. The scale bars are all 8 μm.

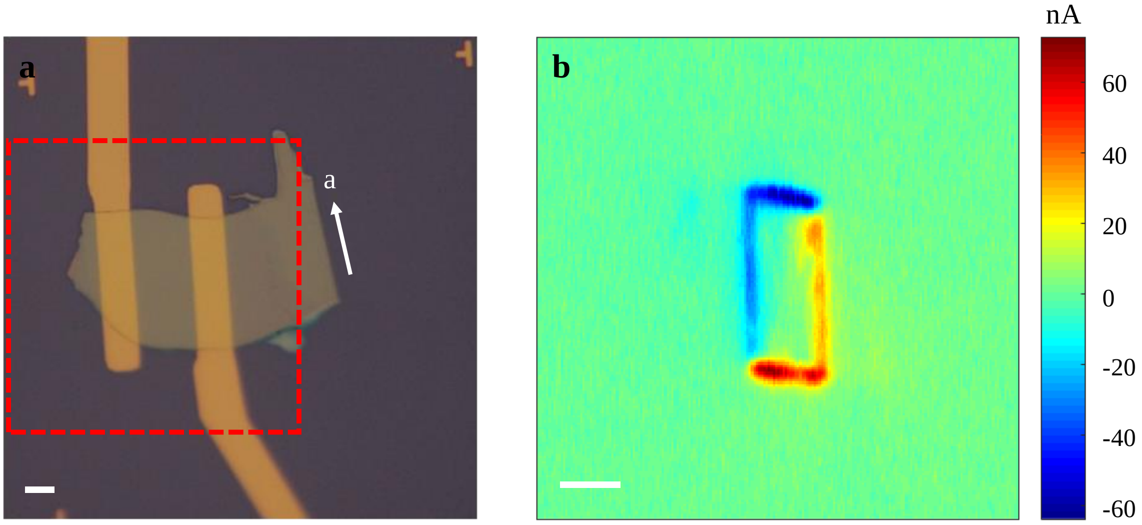

Supplementary Figure 8. Scanning photocurrent microscopy result. (a) Optical microscope image. (b) Scanning photocurrent microscopy result of the rectangular area marked by the red box in Figure a. The measurement was performed with 400-μW 1.96-eV continuum wave excitation. The scale bars are all 10 μm.

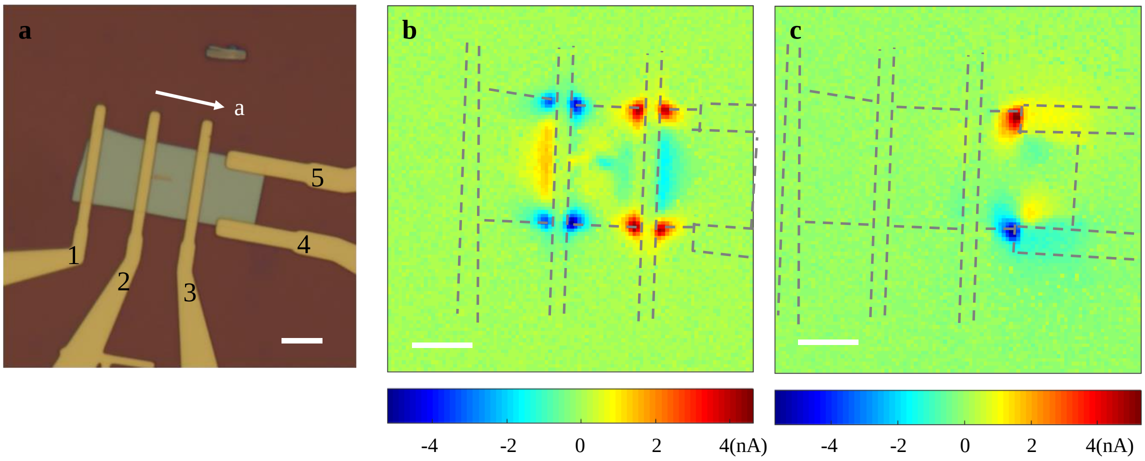

Supplementary Figure 9. Scanning photocurrent microscopy result. (a) Optical microscope image of the WTe_2 device. (b), (c) Scanning photocurrent microscopy results of the photocurrent responses along edges fracture along the crystallographic a-axis (b) and b-axis(c). The measurements were performed using contacts 2 and 3 (b), and contacts 4 and 5 (c), respectively, with 160-μW 1.96-eV pulse excitation. The scale bars are all 10 μm .

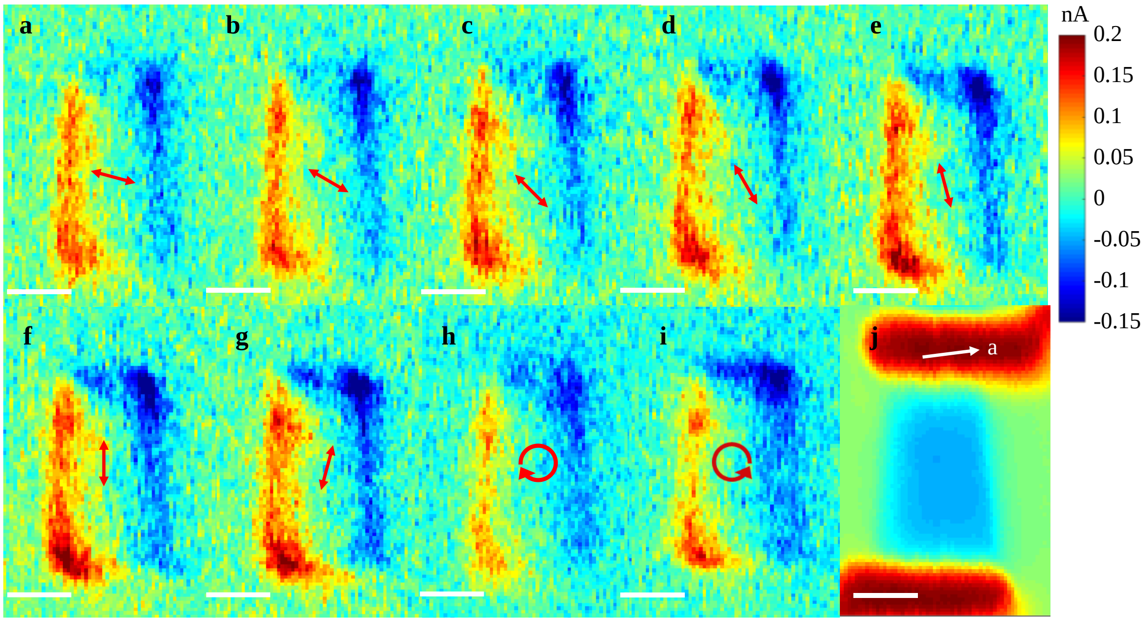

Supplementary Figure 10. Edge photocurrent under different polarization. (a)-(i) Polarization dependence of scanning photocurrent microscopies of the WTe_2 device used in Figure 1 under 10-μW 633-nm CW excitation. The red arrows mark the polarization directions. (j) In-situ scanning reflection microscopy. The scale bars are all 2 μm.

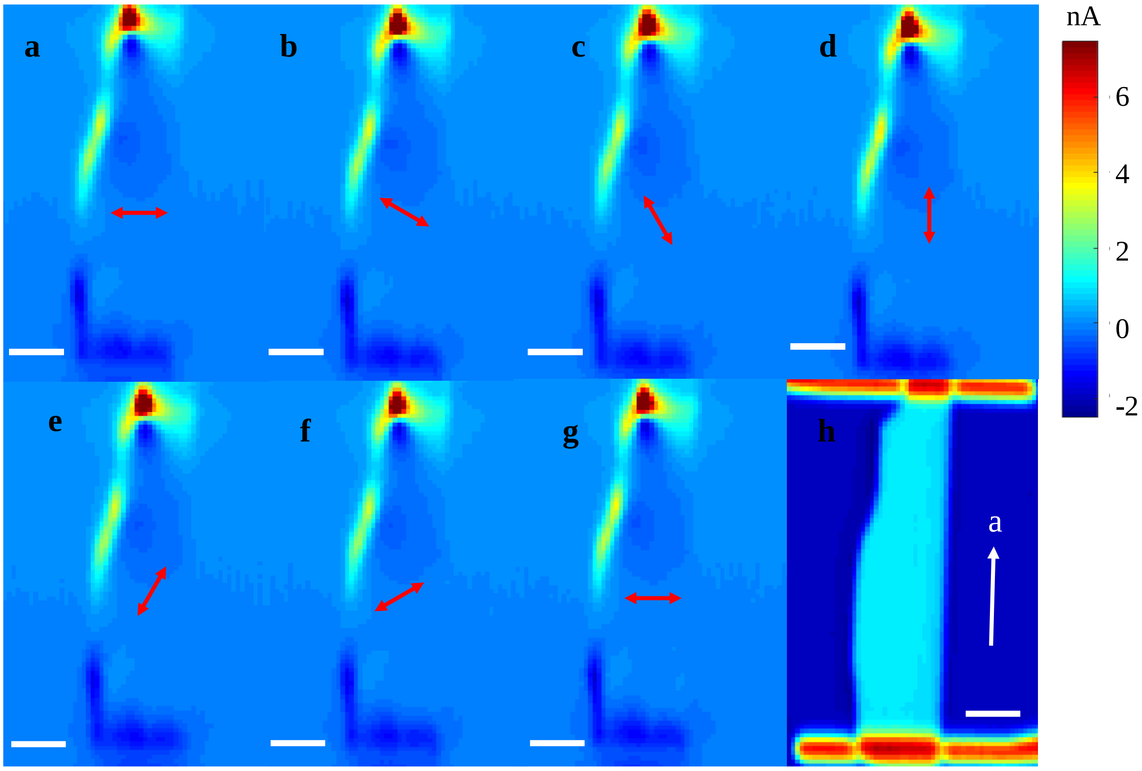

Supplementary Figure 11. Edge photocurrent under different polarization. (a)-(g)

Polarization dependence of scanning photocurrent microscopies of the WTe_2 device used in Figure 2a under 180-μW 633-nm CW excitation. The red arrows mark the polarization directions. (h) In-situ scanning reflection microscopy. The scale bars are all 8 μm.

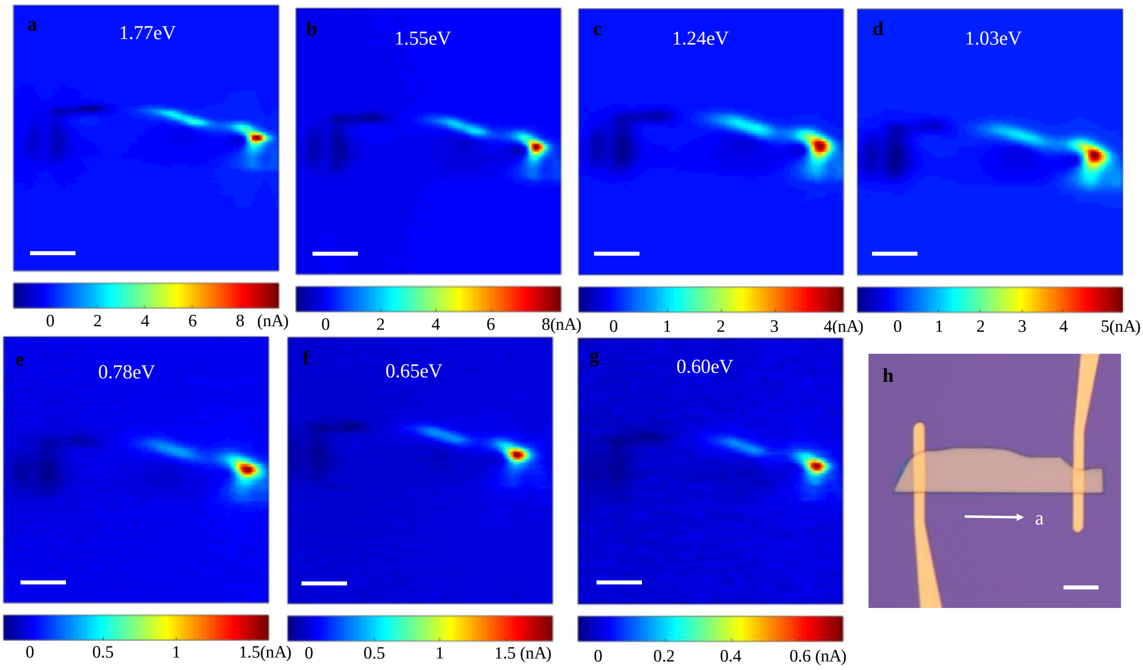

Supplementary Figure 12. Edge photocurrent under different photon energies. (a)-(g) Scanning photocurrent microscopies of the WTe_2 device used in Figure 2a with different excitation photon energies of 180-μW 1.77 eV (a), 180-μW 1.55eV (b), 180-μW 1.24eV (c), 180-μW 1.03 eV (d), 60-μW 0.78eV (e), 50-μW 0.65 eV (f) and 20-μW 0.6 eV (g) respectively; (h) Optical microscope image of the device. The scale bars are all 8 μm.

Supplementary note 1: Polarization resolved Raman spectroscopies of the WTe_2 device.

Polarization resolved Raman spectroscopies were performed to identify the crystallographic axes of the WTe_2 devices. In the Raman measurements, 532-nm CW light was focused onto a ~1 μm spot on the sample, and the excitation polarization dependence of the Raman intensities was obtained by rotating the WTe_2 device with the excitation light polarization fixed. A representative data is presented in Supplementary Figure 4, which was measured on the device used in Figure 2a. Supplementary Figure 4a and b show the Raman spectrums with the excitation polarization parallel and vertical to the long edge of the WTe_2 flake respectively. The

observed Raman peaks are in good agreement with previous Raman scattering studies[1,2]. The intensity of the Raman peak at ~164 cm^{-1} (~212 cm^{-1}) has been reported to reach its maximum (minimum) value when the W-W chains are aligned parallel to the polarization of excitation light. In Supplementary Figure 4c, we have plotted the intensity ratio of these two Raman modes (I_{164} cm^{-1} /I_{212} cm^{-1}), to determine the crystallographic axes, which are labelled in Supplementary Figure 4d.

Supplementary note 2: Scanning photocurrent microscopies of the WTe_2 devices under different excitation polarization and photon energies.

We present more polarization-dependent and photon-energy-dependent scanning photocurrent microscopies (SPCM) results in Supplementary Figure 10, 11 and 12. Supplementary Figure 10 shows SPCM responses of the device used in Figure 1 under different excitation polarization with a fixed power of 10 μW at 633 nm. The output from the laser source went through a Glan Thompson polarizer first to get fixed linear polarized light and then the polarization was controlled by a rotatable half (quarter)-waveplate characterized by an angle θ with respect to the polarization direction of the linear polarizer in a linear (circular) polarization dependent measurement. The response patterns of SPCM show no clear polarization dependence, which indicates the photocurrent did not change directions with different excitation polarization. A slight magnitude dependence on the polarization was observed, which corresponds to anisotropic response and circular photo galvanic effect (CPGE) for linear- and circular-polarization dependent results respectively. Supplementary Figure 11 shows the polarization dependent SPCM results of the device used in Figure 2a with a fixed excitation power of 180 μW at 633 nm. The response patterns show no clear polarization dependence, which indicates the photocurrent does not change the direction under different excitation polarization. Slight magnitude dependence on polarization was observed, which corresponds to anisotropic response. Supplementary Figure 12 shows the SPCM responses of the device used in Figure 2a under different excitation photon energies. Different photon energies were selected from a super-continuum fiber laser by two acousto-optic tunable filter crystals. The response patterns of SPCM show no clear excitation-photon-energy dependence, which indicates the photocurrent did not change directions under different excitation photo energies.

Supplementary References:

1 Lim, S., Rajamathi, C. R., Süß, V., Felser, C. & Kapitulnik, A. Temperature-induced inversion of the spin-photogalvanic effect in WTe_2 and $MoTe_2$. *Physical Review B* **98**, 121301 (2018).

2 Song, Q. *et al.* The In-Plane Anisotropy of WTe_2 Investigated by Angle-Dependent and Polarized Raman Spectroscopy. *Scientific Reports* **6**, 29254 (2016).
